# Research on the Size and Distribution of TiN Inclusions in High-Titanium Steel Cast Slabs

**DOI:** 10.3390/ma18153527

**Published:** 2025-07-28

**Authors:** Min Zhang, Xiangyu Li, Zhijie Guo, Yanhui Sun

**Affiliations:** 1 Collaborative Innovation Center of Steel Technology, University of Science and Technology Beijing, Beijing 100083, China; b20200562@xs.ustb.edu.cn (M.Z.); d202210640@xs.ustb.edu.cn (Z.G.); 2 College of Materials Science and Engineering, Taiyuan University of Technology, Taiyuan 030024, China; 2023520466@link.tyut.edu.cn

**Keywords:** TiN, high-titanium steel, inclusions, continuous cast slabs

## Abstract

High-titanium steel contains an elevated titanium content, which promotes the formation of abundant non-metallic inclusions in molten steel at high temperatures, including titanium oxides, sulfides, and nitrides. These inclusions adversely affect continuous casting operations and generate substantial internal/surface defects in cast slabs, ultimately compromising product performance and service reliability. Therefore, stringent control over the size, distribution, and population density of inclusions is imperative during the smelting of high-titanium steel to minimize their detrimental effects. In this paper, samples of high titanium steel (0.4% Ti, 0.004% N) casting billets were analyzed by industrial test sampling and full section comparative analysis of the samples at the center and quarter position. Using the Particle X inclusions, as well as automatic scanning and analyzing equipment, the number, size, location distribution, type and morphology of inclusions in different positions were systematically and comprehensively investigated. The results revealed that the primary inclusions in the steel consisted of TiN, TiS, TiC and their composite forms. TiN inclusions exhibited a size range of 1–5 µm on the slab surface, while larger particles of 2–10 μm were predominantly observed in the interior regions. Large-sized TiN inclusions (5–10 μm) are particularly detrimental, and this problematic type of inclusion predominantly concentrates in the interior regions of the steel slab. A gradual decrease in TiN inclusion number density was identified from the surface toward the core of the slab. Thermodynamic and kinetic calculations incorporating solute segregation effects demonstrated that TiN precipitates primarily in the liquid phase. The computational results showed excellent agreement with experimental data regarding the relationship between TiN size and solidification rate under different cooling conditions, confirming that increased cooling rates lead to reduced TiN particle sizes. Both enhanced cooling rates and reduced titanium content were found to effectively delay TiN precipitation, thereby suppressing the formation of large-sized TiN inclusions in high-titanium steels.

## 1. Introduction

As a commonly used alloying element, titanium is widely employed in various steels, including microalloyed steels, interstitial-free (IF) steels, and heat-resistant steels [1,2,3,4]. When added to steel, titanium atoms substitute for iron atoms in the iron crystal lattice, causing lattice distortion and impeding dislocation motion, thereby enhancing the strength of the steel through solid solution strengthening. On the other hand, as a strong nitride-forming element, titanium reacts with nitrogen (N) in molten steel to form TiN precipitates. These precipitates inhibit grain growth by pinning grain boundaries, leading to grain refinement [5,6,7] and contributing to dispersion-strengthening and precipitation-strengthening effects [8,9,10].

Since the nitrogen content in steel is generally high, the addition of even small amounts of titanium can fulfil the precipitation conditions for TiN, leading to the formation of TiN inclusions [11,12,13,14]. TiN inclusions in steel typically appear as cubic particles with sharp edges, ranging in size from tens of nanometers to over ten micrometers. Besides existing as individual particles, TiN may also aggregate into large multi-particle clusters. The size of TiN inclusions depends on its precipitation location; those formed during solidification are generally micrometer-scale, while those precipitated in the solid phase are nanometer-scale [15,16,17]. Smaller TiN particles lead to favorable steel properties by refining grains and promoting heterogeneous nucleation [18], whereas larger TiN inclusions, due to their high hardness, non-deformability and sharp edges, can damage the continuity of the steel matrix and become fatigue crack initiation sites under stress, significantly harming steel performance [19]. Fu et al. demonstrated that TiN particles as small as 6 μm can severely reduce steel’s fatigue life [20]. Liu et al. found that when the TiN particle size exceeds 4.9 μm, it induces microcrack propagation and causes toughness fluctuations in Ti-microalloyed steels [21]. Du et al. similarly confirmed that coarse TiN inclusions can serve as cleavage crack initiation sites [22]. Constantino Capurro’s study of medium-carbon steel production revealed that while TiN content is low in molten steel, it increases significantly in continuous cast billets, primarily located in interdendritic regions [23]. Due to the high melting point (2950 °C) of TiN inclusions and their thermodynamically stability, they become hardly removable once formed in steel. Therefore, optimizing smelting processes to mitigate their detrimental effects is crucial during production. Research consistently demonstrates that elemental content and cooling rate predominantly influence TiN precipitation [24,25], and products with elevated Ti and N concentrations promote premature TiN formation, while reducing the content of these elements can delay precipitation. Accelerating cooling rates in the mold effectively suppresses TiN growth. Yan et al. revealed that both the Ti/N ratio and Ti-N concentrations of products affect TiN particle size, recommending maintaining the concentration of elemental products below that of TiN’s solubility product at the solidus temperature with a Ti/N ratio under 3.42, essentially controlling Ti at minimal levels [26]. Tian et al. further established critical Ti/N contents and cooling rates for restricting the TiN particle size to below 6 μm, proposing a three-stage formation mechanism for aggregated TiN [27].

Researchers have investigated the precipitation behavior of TiN inclusions by integrating multiple factors including mass transfer, elemental segregation, and thermodynamics. Ma et al. revealed that in the solidification process of 20 CrMnTi steel containing 0.0072% N and 0.071% Ti, TiN formation occurs only when the solidification fraction exceeds 0.533 [25]. Liu et al. developed a coupled model based on the ChemAppPy platform to investigate the precipitation and growth behavior of TiN inclusions in Ti-containing microalloyed steels. The results demonstrate that, in steel with a 0.06% Ti content, TiN begins to precipitate at a solid fraction of 0.77. Increasing the Ti concentration leads to earlier TiN precipitation. When the cooling rate decreases from 10 K/s to 0.05 K/s, the predicted size of TiN inclusions increases significantly from 0.83 μm to 13.38 μm [28].

In actual production processes, varying cooling rates lead to different precipitation behaviors of TiN at different locations of the slab. Duan et al. found that different cooling rates resulted in significant differences in the average size and number density of TiN between the surface and center of ferritic stainless-steel rolled plates [29]. Que et al. developed an integrated model combining heat transfer, thermodynamic, and kinetic principles to simulate the precipitation and growth of TiN inclusions in steel slabs. The modeling results showed that the average TiN inclusion size increased from 2.82 μm at the slab surface to 10.07 μm at the center. Experimental observations confirmed this trend, demonstrating a progressive decrease in TiN number density accompanied by a corresponding increase in average inclusion size from the surface to the center of the slab [30]. Wang et al. investigated the elemental variations and inclusion distribution in an enameled steel slab. The results revealed that nitrogen content peaked at the slab center and decreased toward the edges. The number density of TiN inclusions exhibited an initial increase followed by a decrease from the inner arc side to the outer arc side, with the largest TiN particle size observed at the slab center [31]. Gao et al.’s research results demonstrate that in titanium-bearing interstitial-free steel slabs, TiN inclusions begin to precipitate when the solid fraction (fs) reaches 0.646–0.68. The TiN–MnS complex inclusions are predominantly concentrated at the quarter-thickness position of the slab [32]. Wang et al. drew similar conclusions in Ti-microalloyed steel slabs, with the peak TiN precipitation occurring at the quarter-thickness position of the slab [33].

Existing studies on TiN inclusion size and precipitation behavior predominantly focus on microalloyed steels, while research on high-titanium steels (with a Ti content significantly exceeding that of microalloyed grades) remains notably scarce. There are even fewer systematic studies on TiN in high-titanium steel slabs. This paper investigates the size distribution of TiN inclusions in high-titanium steel slabs with a titanium content of 0.4% under current production conditions, with a particular focus on the detrimental large-sized TiN inclusions that critically compromise steel matrix integrity and mechanical properties. The analysis further identifies key elemental factors governing TiN growth kinetics, thereby providing theoretical foundations for controlling oversized inclusion formation and suppressing premature TiN precipitation during solidification.

## 2. Experimental Section

### 2.1. Materials

**Figure 1:**
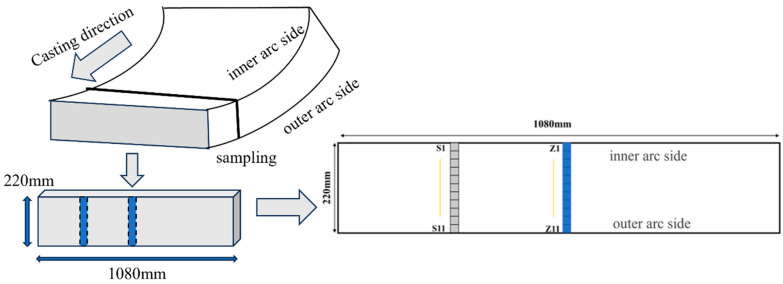
Schematic diagram of casting direction and billet sampling.

The high-titanium steel slabs used in the experiments were obtained from the continuous casting process at a steel plant. The production process of high-titanium steel has the following sequence: converter tapping → ladle refining → RH vacuum refining → continuous casting. To control nitrogen content in the high-titanium steel, an argon-blowing process was implemented in the tundish. Argon supply pipes were installed on the tundish cover, with continuous argon injection at 200 Nm^3^/h during casting to displace air and maintain the atmospheric nitrogen content at below 1% in the tundish. The mold meniscus was protected by argon shielding. The casting speed was maintained between 0.8 and 1.1 m/min. The study involved sampling high-titanium steel cast slabs at two critical locations—the quarter-thickness position (midway between the surface and centerline) and the centerline—with 11 specimens collected from each region along a transverse line spanning from the edge to the center and back to the opposite edge. As shown in Figure 1, the billet quarter-thickness directions from top to bottom are numbered S1–S11, while the center positions are numbered Z1–Z11. The samples were sequentially ground using abrasive paper of varying grit sizes, followed by mechanical polishing with diamond suspensions to achieve a mirror-like finish. The main elemental content of t he high-titanium steel is presented in Table 1.

### 2.2. Analysis

The Reaction module of thermodynamic software Fact Sage 8.1 was employed to calculate the Gibbs free energy changes in three the titanium-containing inclusions (TiN, TiS and TiC) in the high-titanium steel within the temperature range of 1000 °C to 1600 °C, using the FactPS and FToxid databases.

The Particle X automated inclusion analysis system was used to analyze the size, composition, and number of inclusions in the steel, with an initial detection threshold of 1 μm and a scanning area of about 55 mm^2^ per sample to ensure statistical reliability. The bulk composition of major alloying elements was detected using chemical ICP-AES methods, with a minimum accuracy of 5 ppm, while oxygen (O) and nitrogen (N) contents were measured using an inert gas fusion-based oxygen/nitrogen analyzer (TCH600).

## 3. Result and Discussion

### 3.1. Inclusion Analysis in Slab

#### 3.1.1. Typical Inclusions in Slab

**Figure 2:**
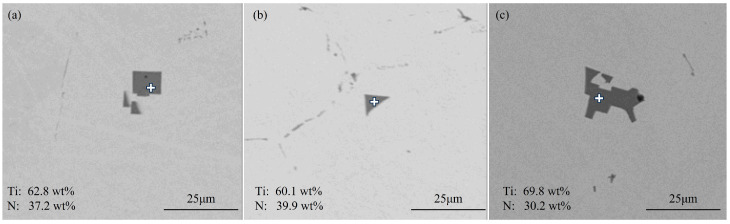
Typical TiN inclusions at the quarter-thickness position of the slab: (**a**) cubic particles with sharp edges; (**b**) triangular particles with angular facets; (**c**) clustered.

Figure 2 shows typical TiN inclusions at the quarter-thickness position of the slab. The inclusions exhibit three distinct morphologies: Figure 2a shows cubic particles with sharp edges; this TiN inclusion measured 8.7 μm in size, and this type of TiN inclusion was the most commonly observed in the slab. Figure 2b shows triangular particles with angular facets. Figure 2c shows large aggregated inclusions formed by the clustering of individual TiN particles, as evidenced by the preserved sharp edges and relatively regular morphology at the boundaries of these aggregates. This type of inclusion typically measures approximately 20 μm in size.

**Figure 3:**
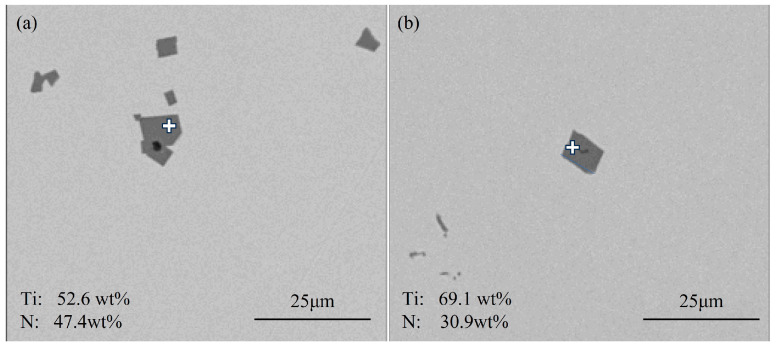
Typical TiN inclusions at the center of the slab: (**a**) agglomerated; (**b**) angular morphology.

Figure 3 shows typical TiN inclusions at the center position of the slab. Figure 3a shows agglomerated large TiN inclusions measuring 8.07 μm with sharp edges. Figure 3b shows isolated cubic TiN particles measuring 7.36 µm showing a similar angular morphology. No morphological differences were observed between TiN inclusions at the center and quarter-thickness positions, with variations only in size distribution.

**Figure 4:**
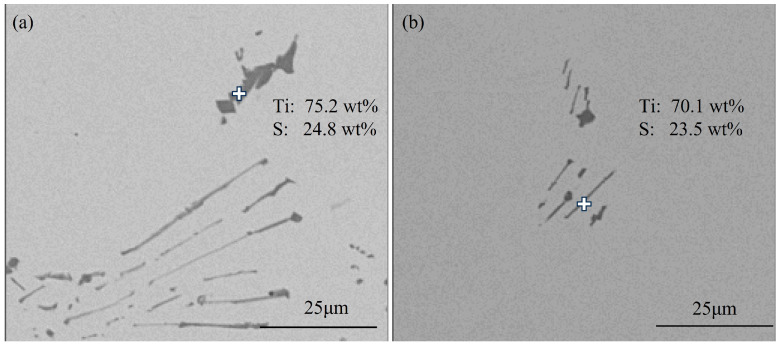
Typical TiS inclusions at the quarter-thickness position of the slab: (**a**) irregular morphologies; (**b**) aggregated short rod-shaped.

Figure 4 shows typical TiS inclusions at the quarter-thickness position of the slab. Most TiS inclusions appeared as aggregated short rod-shaped particles, as shown in Figure 4b, while some agglomerated TiS inclusions exhibited irregular morphologies, as illustrated in Figure 4a.

**Figure 5:**
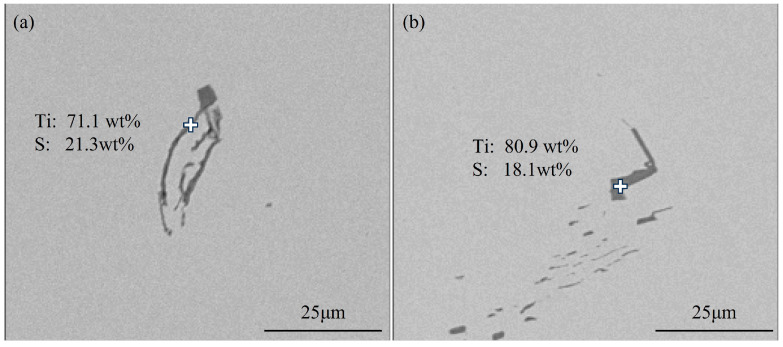
Typical TiS inclusions at the center of the slab: (**a**) short rod-shaped forms; (**b**) irregular morphologies.

Figure 5 shows typical TiS inclusions at the center position of the slab. The TiS morphologies at the center of slab closely resemble those at the quarter-thickness position, primarily exhibiting elongated rod-shaped forms, as can be seen in Figure 5a, or short rod-shaped forms, along with irregular morphologies, as demonstrated in Figure 5b.

**Figure 6:**
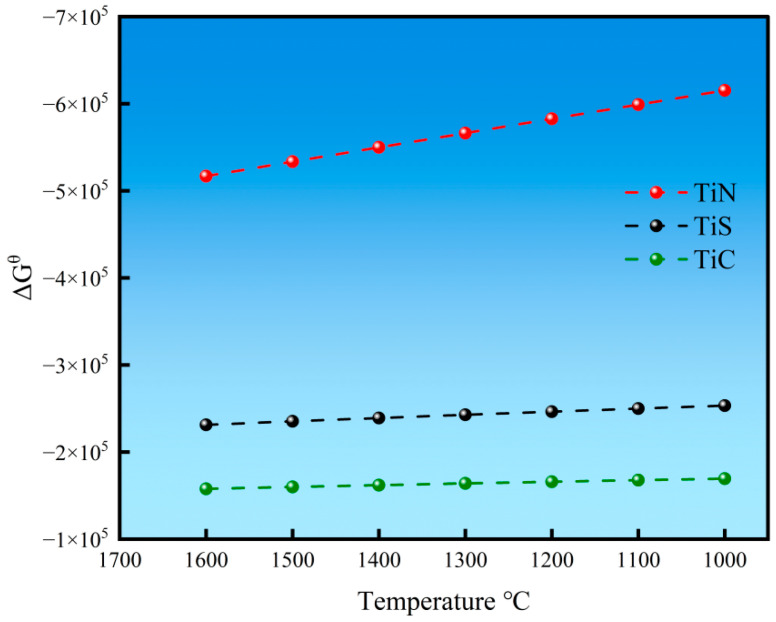
Gibbs free energy of titanium-containing inclusions.

The calculation results of the Gibbs free energy changes in the three titanium-containing inclusions are shown in Figure 6. According to the calculation results, at 1600 °C, the Gibbs free energy of all three types of inclusions was negative, indicating that under thermodynamic conditions, all three inclusions could precipitate. Among them, TiN consistently exhibited the lowest Gibbs free energy, suggesting that TiN inclusions are more likely to form. Furthermore, as the temperature decreased, the Gibbs free energy of TiN continued to decrease, demonstrating that the precipitation of TiN would persist.

#### 3.1.2. Inclusion Distribution Across Slab Thickness

**Figure 7:**
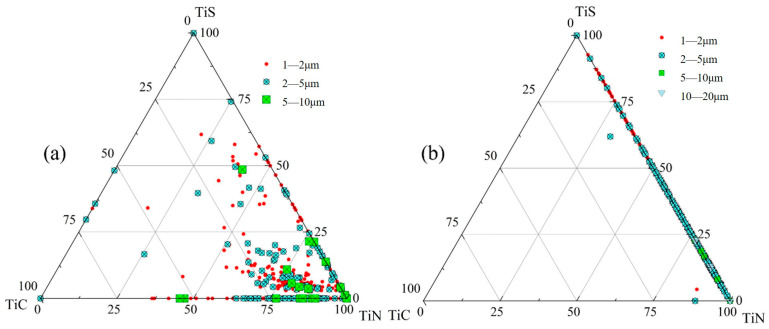
Inclusion distribution map of edge samples: (**a**) S1; (**b**) Z1.

Figure 7 presents inclusion distribution maps and a size-classified composition analysis of inclusions from edge samples at both the quarter-thickness and center positions of the slab. Figure 7a applies to S1 and Figure 7b applies to Z1. For each sampling location, 1000 randomly selected inclusions were characterized and categorized by size into five ranges: 1–2 μm, 2–5 μm, 5–10 μm, 10–20 μm, and >20 μm. At the edge region of the slab’s quarter-thickness position, the composition of most inclusions falls within the lower right corner of the diagram. This indicates that, apart from single-phase TiX (X = C,N,S) inclusions, the composite inclusions primarily consist of Ti (C,N) and TiN + TiS, with TiN being the dominant component in these complex inclusions. A small number of inclusions have compositions corresponding to TiS or TiC, with sizes ranging from 1 to 5 µm. In this sample, the inclusion sizes are mainly distributed in two ranges: 1–2 μm and 2–5 μm, with a minority of inclusions measuring 5–10 μm. Notably, these larger inclusions (5–10 μm) also exhibit compositions located in the lower right corner of Figure 7a.

The edge sample from the slab center exhibits inclusions, most measuring 2–5 μm, with their compositional data points almost exclusively distributed along the TiN-TiS phase line. At this location, apart from single-phase TiN and TiS inclusions, nearly all other inclusions consist of TiN + TiS composites.

**Figure 8:**
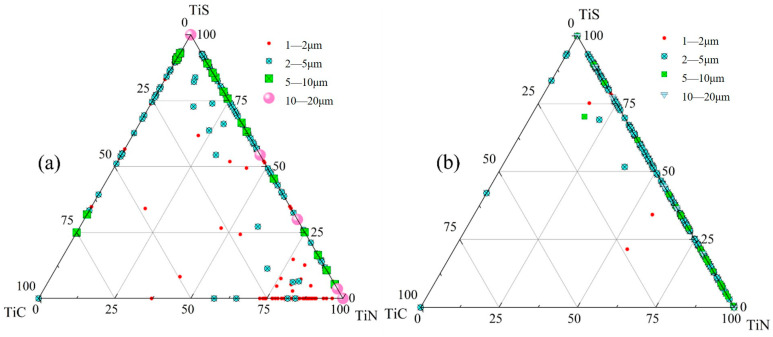
Inclusion distribution map of interior samples: (**a**) S3; (**b**) Z3.

The compositional distributions of inclusions in samples S3 and Z3 are presented in Figure 8. In sample S3 from the quarter-thickness of the slab, the compositional data points of complex inclusions are mainly distributed along the three edges of the ternary phase diagram. In this sample, apart from single-phase TiX (X = C,N,S) inclusions, the composite inclusions primarily consist of TiC + TiS and TiN + TiS, with a small amount of Ti (C,N), and the sizes of these inclusions are predominantly in the range of 1–2 μm. The TiC + TiS inclusions are predominantly 2–5 μm in size and contain a relatively higher TiS content. The TiN + TiS inclusions include a few large particles exceeding 20 μm.

In sample Z3 from the slab center, the inclusion composition points are primarily distributed at the TiN vertex, at the TiS vertex and along the TiS−TiN line, with a small number of single-phase TiC and TiC + TiS composite inclusions present. In this sample, the 2–5 μm inclusions mainly consist of TiS, TiN and TiS + TiN, along with minor amounts of TiS + TiC and TiC. The composition points of 5–10 μm inclusions are predominantly located near the TiN side of the TiS-TiN line. All 10–20 μm inclusions are situated along the TiS−TiN line, with compositions of TiN, TiS and TiS + TiN.

**Figure 9:**
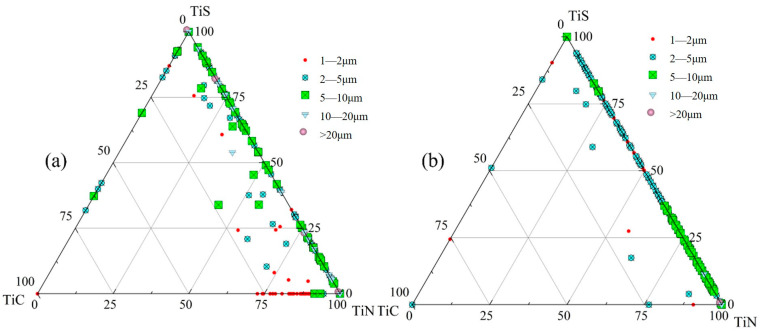
Inclusion distribution map of center samples: (**a**) S6; (**b**) Z6.

The compositional distributions of inclusions in the centerline samples (S6 and Z6) from both the slab center and quarter-thickness positions are presented in Figure 9. At the center of the quarter-thickness position in the slab (S6), the compositional data points of inclusions are predominantly located along the TiS + TiN boundary, indicating that the primary inclusion phases in this sample are TiS + TiN composites. A minor fraction of data points are distributed along the TiS + TiC and TiC + TiN boundaries. Most 1–2 μm inclusions exhibit compositions clustered near the TiN side of the TiC-−TiN boundary. For 10–20 μm inclusions, while some data points correspond to pure TiS and TiN at the vertices, the majority align along the TiS + TiN line, confirming their composition as TiN, TiS, and TiN + TiS phases. The relatively few inclusions exceeding 20 μm in size show compositional coordinates either at the TiS/TiN vertices or along the TiS-−TiN boundary, demonstrating that these large inclusions consist exclusively of TiN, TiS, and TiN + TiS.

In sample Z6 from the slab center, the compositional data points of the inclusions are predominantly distributed along the TiS − TiN phase boundary. Specifically, the 5–10 μm inclusions are mainly concentrated on the TiN side of the TiS-TiN line, while the 10–20 μm inclusions are exclusively located on the TiN-rich side, with TiN content exceeding 90%. A small number of larger inclusions (>20 μm) are positioned closest to the TiN vertex, demonstrating a clear trend of increasing TiN content with inclusion size.

In this experiment, TiS, TiC, and TiN inclusions, as well as their composite inclusions, were observed. Choi [34] and Li [35], respectively, demonstrated that both TiN and TiS exhibit a pinning effect capable of inhibiting grain growth. TiS inclusions precipitate during solidification [36], and both TiS and Ti4 C_2_ S_2_ inclusions demonstrate good pitting resistance, with minimal impacts on the mechanical properties of steel [37]. TiC precipitates in the solid phase. As shown in Figure 8 and Figure 9, in the high-titanium steel used in this experiment, the quantity of individual TiC and TiC-containing inclusions was significantly lower than that of TiN. Among the three types of titanium-containing inclusions, TiN has the most significant influence on steel performance and service life. Fine TiN particles can pin grain boundaries and inhibit grain coarsening, while large-sized TiN inclusions disrupt matrix continuity, leading to microcrack formation and deteriorating processing performance. Therefore, the following analysis focuses on the size distribution and precipitation behavior of TiN inclusions in steel.

### 3.2. Size Distribution of TiN Inclusions

**Figure 10:**
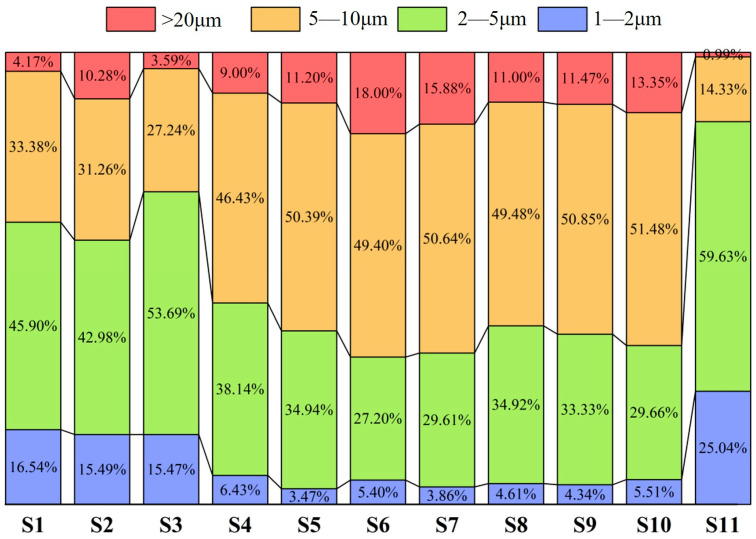
Size distribution of TiN inclusions at quarter-thickness position of slab.

**Figure 11:**
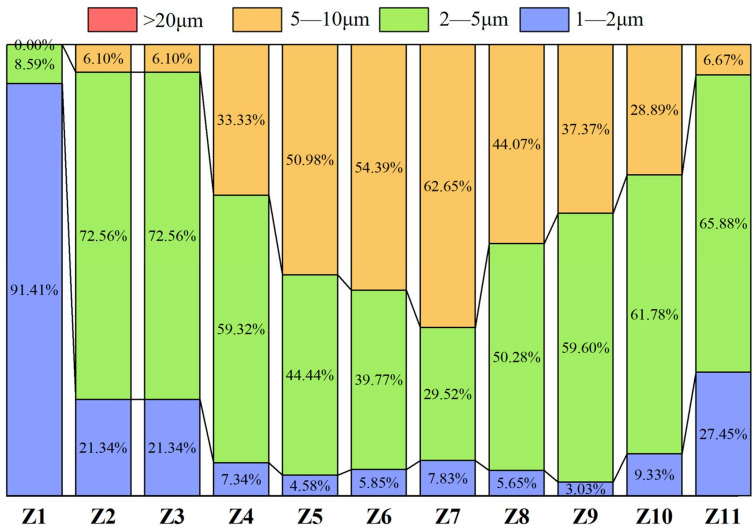
Size distribution of TiN inclusions at slab center.

According to the statistical results of TiN inclusion sizes in samples from the quarter-thickness and center positions of the slab, Figure 10 and Figure 11 were plotted. Figure 10 shows the size distribution of TiN inclusions at the quarter-thickness position of the slab, while Figure 11 presents the size distribution of TiN inclusions at the center position. The statistical results indicate that at the quarter-thickness position of the slab, the size of TiN inclusions is primarily in the range of 2–10 μm, whereas at the center position, TiN inclusions are all within 1–10 μm, with no large inclusions exceeding 10 μm.

A study by Que et al. [30]. demonstrated that in microalloyed steel containing 0.12% Ti, TiN inclusions were predominantly concentrated in the 0–2 μm and 2–4 μm size ranges, accounting for 33% and 20% of the total population, respectively. In contrast, larger TiN inclusions (6–8 μm and 8–10 μm) showed significantly lower proportions, each constituting less than 10%. The TiN distribution in Figure 10 and Figure 11 is predominantly concentrated in the 2–10 μm range. This discrepancy with Que et al.’s results can be partially attributed to the higher Ti content (0.4% vs. 0.12%) in our high-titanium steel, which promoted earlier TiN precipitation and consequently led to larger inclusion sizes.

As shown in Figure 10, at the quarter-thickness position, 1–2 μm TiN inclusions accounted for a relatively high proportion near the edge, reaching 16.54% (S1) and 25.04% (S11). This is because the cooling rate near the edge was higher than that in the interior, leading to the precipitation of smaller TiN inclusions. This faster solidification limited the growth time, resulting in a higher proportion of fine TiN inclusions compared to that in the interior. In the inner samples, the proportion of 1–2 μm inclusions decreased to around 5%, with the lowest values observed in S5 (3.47%) and S7 (3.86%). For 2–5 μm TiN inclusions, the distribution trend was similar—the proportion was higher in edge samples, reaching 45.90% (S1) and 59.63% (S11). In the inner samples, the proportion decreased to approximately 35%, with the lowest values observed in S6 (27.20%) and S7 (29.61%). The distribution of 5–10 μm TiN inclusions followed an inverted “V” pattern, with a lower proportion near the edge (33.38% in S1 and 14.33% in S11) and a gradual increase toward the interior, reaching around 50%. Among the six samples from S5 to S10, the proportion of TiN inclusions with sizes ranging from 2 to 5 μm reached its maximum value, at approximately 50%. Additionally, a certain proportion of large TiN inclusions (>10 μm) were present at the quarter-thickness position. These large inclusions were formed by the aggregation of single-phase TiN particles. Their distribution trend was similar to that of 5–10 μm inclusions—the proportion was lowest near the edge (0.99% in S11) and increased toward the interior, reaching a maximum of 18%.

As shown in Figure 11, at the center position of the slab, the proportion of 1–2 μm TiN inclusions was relatively high in the edge samples, reaching 91.41% (Z1) and 27.45% (Z11). During the continuous casting process, water accumulation on the inner arc side resulted in asymmetric cooling intensity between the inner and outer arcs. The Z1 position located on the inner arc side experienced faster cooling rates, which promoted the precipitation of fine inclusions. This phenomenon may explain the anomalous abundance of small-sized (1–2 μm) TiN inclusions observed at the Z1 location. In the interior samples, the proportion of 1–2 μm inclusions exhibited a continuous decline, reaching its minimum value of 3.03% at Z9, similarly to the distribution observed at the quarter-thickness position. For 2–5 μm inclusions, the distribution exhibited a distinct “V” shape. Except for Z1, this size range dominated in the edge samples, accounting for 72.56% (Z2, Z3) and 65.88% (Z11). Toward the interior, the proportion gradually decreased, reaching a minimum of 29.52% (Z7) at the center. In contrast, 5–10 μm inclusions showed a reverse trend—they accounted for only 6.67% (Z11) at the edge but increased in concentration significantly in the interior, peaking at 62.65% (Z7) at the center.

**Figure 12:**
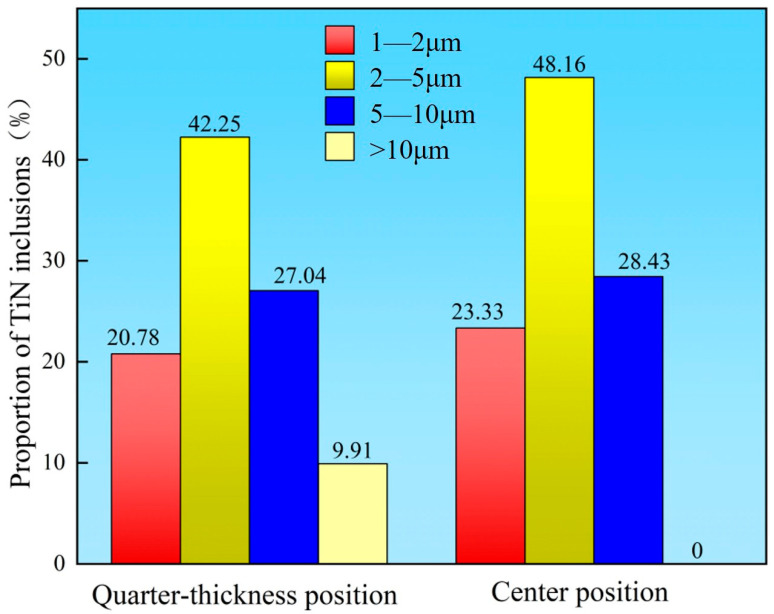
Size distribution of TiN inclusions.

Statistical analysis and comparison of the total size distribution of TiN inclusions were conducted for both the quarter-thickness position and center position of the slab. Through statistical analysis of the total TiN inclusions at both the slab center and quarter-thickness positions, along with the quantity of TiN inclusions within each size range, the proportional distribution of differently sized TiN inclusions at these two locations was determined. The size distributions of TiN inclusions at these two locations showed similar characteristics. As shown in Figure 12, inclusions in the 2–5 μm range accounted for the highest proportion, representing 42.25% of the inclusions at the quarter-thickness position and 48.16% at the center position. The second most prevalent size range was 5–10 μm, constituting 27.04% at the quarter-thickness position and 28.43% at the center. Inclusions measuring 1–2 μm represented 20.78% and 23.33% of the inclusions at the quarter-thickness and center positions, respectively. Notably, the quarter-thickness position contained an additional 9.91% of TiN inclusions larger than 10 μm, while no such large inclusions were observed at the slab center.

**Figure 13:**
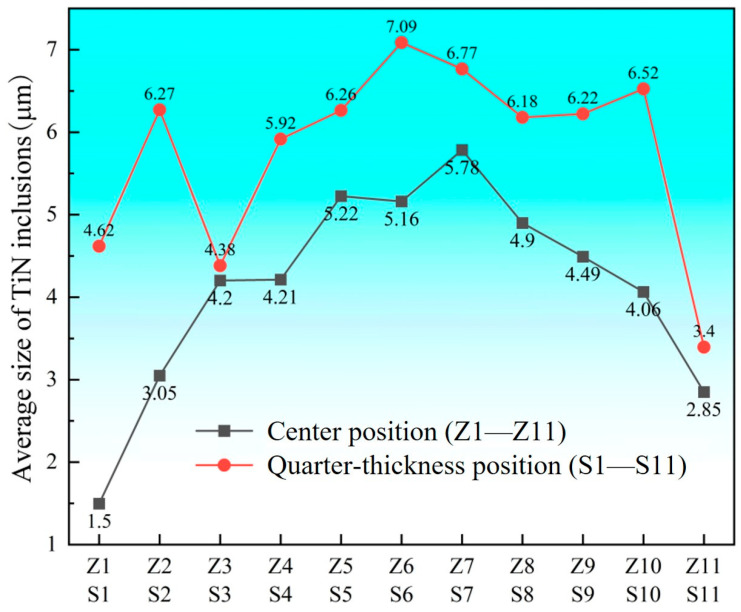
Average size of TiN inclusions at slab position and quarter-thickness position.

Figure 13 presents the average sizes of TiN inclusions across different sampling positions at both locations. With the exception of edge samples at the center position, which showed smaller average sizes, inclusion dimensions at all other sampling positions generally ranged between 3 and 7 μm. Both locations exhibited an inverted “V” distribution pattern of average inclusion sizes: as sampling positions progressed from the edge toward the interior, the average size gradually increased, reaching maximum values of 7.09 μm (quarter-thickness position) and 5.78 μm (center position), respectively. Notably, the average inclusion sizes at the quarter-thickness position consistently exceeded those at the center position throughout all comparable sampling locations.

### 3.3. Number Density of TiN Inclusions

**Figure 14:**
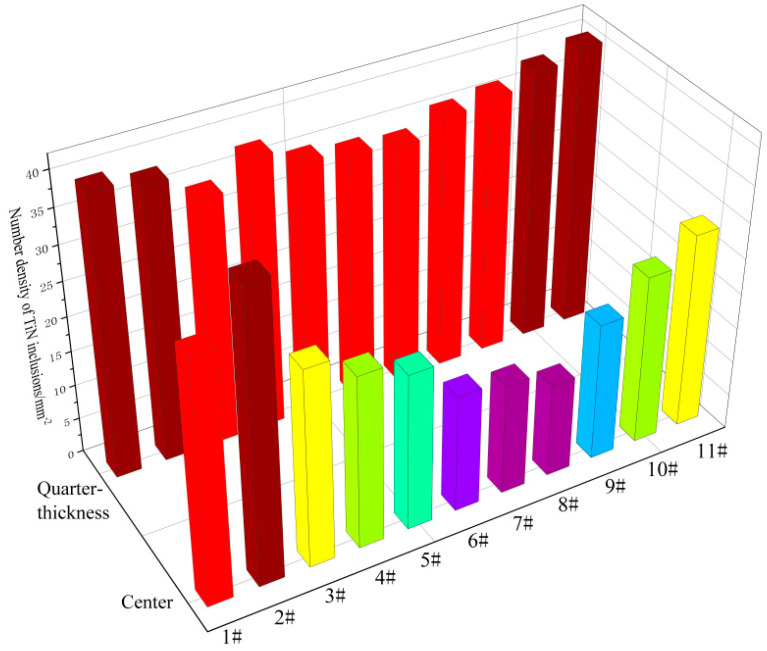
Variation in TiN inclusion content.

The number density of TiN inclusions in each sample in the quarter-thickness position and center position of the slab was statistically analyzed based on the count of TiN-containing inclusions within the scanned sample area and the scanning area size. As for Figure 14, a color-gradient map was generated to visualize the number density of TiN inclusions, where the color scale ranges from purple to red, indicating increasing number density (purple represents a low number density and red represents a high number density); the lowest number density is 12%, and the highest number density is 42%. Comparative analysis of the color patterns reveals distinct differences in TiN distribution between the quarter-thickness position and center position. The number density of TiN inclusions at the quarter-thickness position of the slab is generally higher than that at the center of the slab. The number density of TiN inclusions shows a consistent variation trend at both locations, with higher values observed in the edge samples and a gradual decrease observed in the interior samples. The most central sample exhibits the lowest number density of TiN inclusions. At the quarter-thickness position of the slab, the edge samples exhibited the highest TiN inclusion number densities of 40.05 mm^−2^ (S1) and 40.3 mm^−2^ (S11), while the lowest recorded density was 34.2 mm^−2^ (S7). At the center position of the slab, the edge samples showed the highest TiN inclusion number density, with samples Z1 and Z2 measuring 34.5 mm^−2^ and 41.3 mm^−2^, respectively. The corresponding opposite specimens (Z10 and Z11) exhibited significantly lower inclusion densities of 24.2 mm^−2^ and 27.0 mm^−2^, respectively, compared to Z1 and Z2. Notably, the three interior specimens (Z6–Z8) showed the lowest TiN inclusion densities, measuring 16.8 mm^−2^ (Z6), 15.8 mm^−2^ (Z7), and 13.4 mm^−2^ (Z8), respectively. Based on the statistical results, the number density of TiN inclusions at the quarter-thickness position of the slab was higher than that at the center position, indicating the presence of a greater quantity of TiN inclusions at the quarter-thickness location. The number density at the edge region was higher than that in the interior. This phenomenon may be attributed to the fact that the central region experienced the final solidification stage with a reduced temperature gradient, leading to a decreased nucleation driving force for TiN inclusions. Therefore, the number density of TiN inclusions gradually decreased from the slab edge to the interior.

Both Que and Duan quantitatively analyzed the variations in TiN inclusion number density within Ti-microalloyed steel and Ti-stabilized ultra-pure ferritic stainless steel slabs [29,30], respectively. Their findings align with our experimental results, demonstrating a gradual decrease in inclusion number density from the surface layer to the interior region. This phenomenon is attributed to the fact that the slab surface had the fastest cooling rate and shortest solidification time.

### 3.4. Calculation of TiN Inclusion Precipitation

#### 3.4.1. TiN Precipitation Temperature

The standard Gibbs free energy (ΔG^θ^) for TiN precipitation is calculated as follows [29]:

(1) [Ti]+[N]=TiN(s)

(2) $$\Delta G^{\theta }=-314800+114.35\cdot T$$

(3) $$\Delta G=\Delta G^{\theta }+RTlnK$$

(4) $$K=\frac{a_{TiN}}{a_{\left[N\right]}\cdot a_{\left[Ti\right]}}=\frac{a_{TiN}}{\left(f_{\left[N\right]}\cdot w_{\left[N\right]})\cdot (f_{\left[Ti\right]}\cdot w_{\left[Ti\right]}\right)}$$

In Equation (4), a denotes the activities of products and reactants, and w denotes the content of each element, with w_[Ti]_ and w_[N]_ being 0.4% and 0.002%, respectively. f is the activity coefficient; the activity coefficient f of each element in molten steel can be expressed in terms of the interaction coefficients, e, and the elemental content, w, as shown in Equation (5):

(5) $$lgf_{i}=\sum_{j=1}^{n}e_{i}^{j}\cdot w_{\left[j\right]}$$

By substituting the elemental contents from Table 1 and the interaction coefficients from Table 2 into Equation (5), the activities of nitrogen (a_[N]_) and titanium (a_[Ti]_), respectively, in the system were obtained To simplify the calculation, the activity of TiN (a_TiN_) was set as 1, while the activity coefficients and contents of Ti and N were incorporated into Equation (4). When ΔG = 0 in Equation (3), the calculated precipitation temperature of TiN was determined to be 1829.1 K (1556 °C).

The calculation formulas for the liquidus and solidus temperatures are given by Equations (6) and (7), respectively:

(6) $$T_{L}=1536-65w_{\left[C\right]}-30w_{\left[P\right]}-25w_{\left[S\right]}-20w_{\left[Ti\right]}-8w_{\left[Si\right]}-5w_{\left[Mn\right]}-2.7w_{\left[Al\right]}-90w_{\left[N\right]-}2w_{\left[Mo\right]}$$

(7) $$T_{S}=1538-175w_{\left[C\right]}-280w_{\left[P\right]}-575w_{\left[S\right]}-40w_{\left[Ti\right]}-20w_{\left[Si\right]}-30w_{\left[Mn\right]}-7.5w_{\left[Al\right]}-5w_{\left[Mo\right]}$$

Substituting the elemental content of high-titanium steel into Equations (6) and (7), the liquidus temperature that can be obtained is 1431.37 °C (1704.5 K) and the solidus temperature is 1506.12 °C (1779.2 K). The precipitation temperature of TiN is higher than the liquidus temperature, indicating that in a high-titanium steel system with 0.4% Ti and 0.004% N, TiN inclusions begin to precipitate in the liquid phase. Assuming the liquidus temperature remains constant at 1779.2 K and the nitrogen content in the molten steel is unchanged, the critical titanium content for TiN precipitation at the liquidus temperature is approximately 0.17%. Only when the Ti content is below 0.17% will TiN precipitate in the two-phase region. Similarly, assuming the solidus temperature remains at 1704.5 K with a constant nitrogen content, the critical titanium content for TiN precipitation at the solidus temperature is about 0.08%, and TiN will only precipitate in the solid-phase region when the Ti content falls below 0.08%. Comparing the critical Ti contents at different precipitation temperatures reveals that the precipitation temperature of TiN increases with a higher Ti content in the system. This demonstrates that increasing Ti content promotes earlier TiN precipitation, while reducing Ti content delays its formation.

During the solidification of molten steel, the segregation of solute elements leads to the enrichment of Ti and N in the liquid phase. This results in localized concentration products exceeding the equilibrium threshold required for TiN precipitation, thereby causing premature TiN formation. Consequently, the redistribution of solute elements (Ti and N) between the two phases must be considered. According to the law of mass conservation, the initial solute concentration (C_0_) in the steel melt equals the sum of solute contents in both liquid and solid phases.

(8) $$C_{0}=f_{s}\cdot C_{s}+\left(1-f_{s}\right)\cdot C_{L}$$

The solute partition coefficient (K) can be expressed as follows:

(9) $$K=\frac{C_{S}}{C_{L}}$$

By substituting the solute partition coefficient K into Equation (8), the relationship between solute content in the liquid phase, C_L_, and the solidification parameter, f, is obtained, as shown in Equation (10)

(10) $$C_{L}=\frac{C_{0}}{1+f_{s}\;\left(K-1\right)}$$

**Figure 15:**
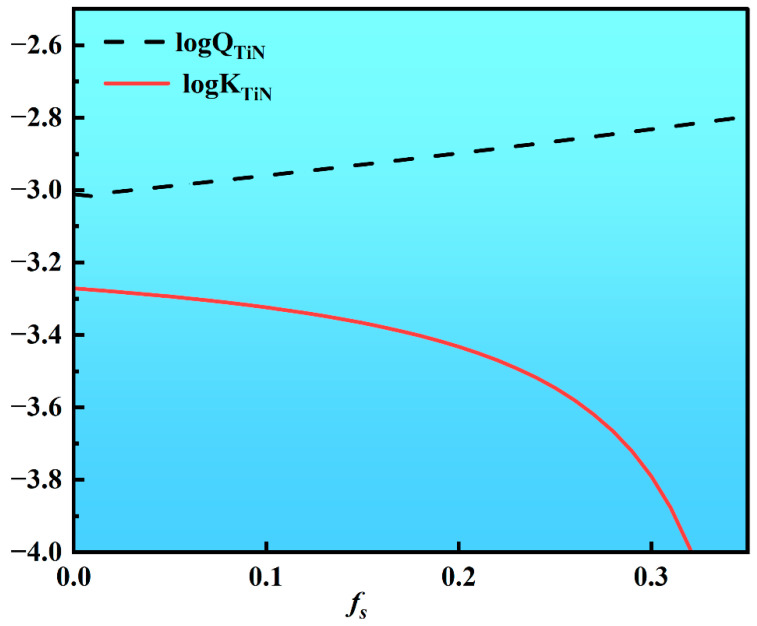
The relationship between the Q_TiN_ and K_TiN_ values of TiN precipitation with varying solid fractions.

During solidification, the activity product (Q_TiN_) for TiN precipitation in the two-phase region is expressed as follows:

(11) $$Q_{TiN}=\left(f_{Ti}\cdot w_{Ti}\right)\cdot \left(f_{N}\cdot w_{N}\right)$$

f_Ti_ and f_N_ represent the activity coefficients of Ti and N, respectively, with the initial contents of w_0 [Ti]_ = 0.4% and w_0[N]_ = 0.004%. Substituting these parameters into the equation yields the relationship between the actual activity product, Q_TiN_, and the solid fraction, fs. As shown in Figure 15, the actual activity product for TiN precipitation (Q_TiN_) already exceeds the equilibrium activity product at the onset of solidification (fs = 0), indicating that TiN begins precipitating in the liquid phase prior to solidification. During solidification, the actual activity product Q_TiN_ continuously increases with progressive solid fraction (fs), persistently remaining above the equilibrium value throughout the process, thereby driving continuous TiN precipitation.

#### 3.4.2. Calculation of TiN Inclusion Precipitation Size

The calculation formula for the precipitation radius of TiN inclusions during steel solidification is as follows [13]:

(12) $$r\cdot \frac{dr}{dt}=\frac{M_{TiN}\cdot \rho_{Fe}}{100M_{Fe}\cdot \rho_{TiN}}\cdot D_{N}\cdot (w_{N}-w_{\mathrm{eqN}})$$

r is the precipitation radius of TiN inclusions (in cm); t is solidification time; M_TiN_ is the molar mass of TiN (62 g/mol); M_Fe_ is the molar mass of steel (taken as 56 g/mol, Fe’s atomic mass); ρ_TiN_ is the density of TiN inclusions (5.43 g/cm^3^); ρ_Fe_ is the density of molten steel (7.07 g/cm^3^); D_N_ is the diffusion coefficient of nitrogen (N) in molten steel; w_N_ is the mass fraction of N at the solidification front; w_eqN_ is the equilibrium mass fraction of N at the onset of TiN precipitation. By using the following equation, the relationship between the precipitation radius r of TiN inclusions and the cooling rate can be derived.

(13) $$r=\sqrt{\frac{M_{TiN}\cdot \rho_{Fe}}{50M_{Fe}\cdot \rho_{TiN}}D_{N}\cdot (w_{N}-w_{\mathrm{eqN}})\cdot \tau }$$

The calculation formula for the local cooling time, τ, is as follows:

(14) $$\tau =\frac{T_{L}-T_{S}}{R_{C}}$$

**Figure 16:**
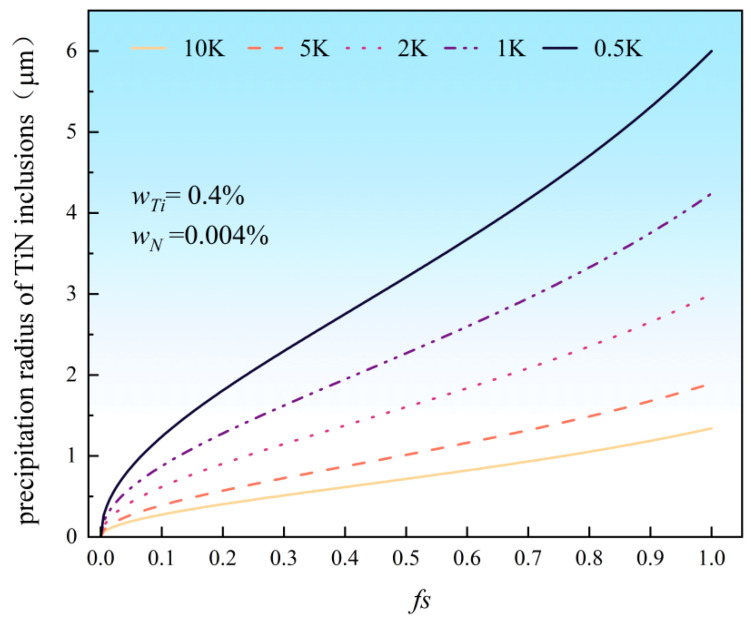
Relationship between TiN precipitation size and solid fraction under different cooling rates.

The relationships between the precipitation radius, r, of TiN inclusions and the solid fraction, fs, were computed for Rc = 10, 5, 2, 1, and 0.5. The adopted range (0.5–10 K/s) encompasses all probable cooling rates [41,42,43]. As shown in Figure 16, the results indicate that the precipitation radius of TiN decreases with increasing cooling rate. At the minimum cooling rate of 0.5 K/s, the maximum radius reaches approximately 6 μm, whereas at the maximum rate of 10 K/s, it reduces to only 1.3 μm. When R_C_ > 2 K/s, the maximum radius remains below 2 μm, with minimal impact from further rate increases. Below 2 K/s, the radius increases significantly as the rate decreases. Therefore, controlling the cooling rate is an effective approach to refine TiN inclusion sizes when the Ti and N contents in molten steel are fixed.

## 4. Conclusions

1. The primary inclusions in high-titanium steel consist of TiN, TiS, TiC and their composite forms. At the quarter-thickness position of the slab, inclusions demonstrate distinct size–composition correlations: small-sized inclusions (1–2 μm) are predominantly Ti (C,N) and TiN + TiS; medium-sized inclusions (2–10 μm) and large inclusions (>10 μm) are mainly TiS, TiN and their composite phases. Edge samples show TiN-rich characteristics, whereas interior and central regions exhibit increasing TiS content. The overall pattern reveals increasing TiN content with inclusion size growth. The inclusions in the central region of the slab are predominantly composed of TiN, TiS, and their composite phase TiN + TiS. The inclusion sizes are mainly distributed in the 2–10 μm range, exhibiting a distinct size–composition correlation: larger inclusions (greater than 5 μm) show higher TiN content, while the 2–5 μm TiS-TiN inclusions contain a relatively higher TiS composition. This size-dependent compositional variation demonstrates a progressive enrichment of TiN with increasing inclusion size.
2. At the quarter-thickness position of the slab, TiN inclusions primarily range between 2 and 10 μm, with a small number of larger inclusions exceeding 10 μm. In contrast, at the slab center, TiN inclusions are mainly 1–5 μm in size, with no large-sized TiN inclusions observed. Due to cooling rate effects, samples from the edge regions at both positions exhibit a higher proportion of smaller TiN inclusions, whereas interior samples show a predominance of larger-sized TiN inclusions. Within the interior regions of the steel slab, a substantial proportion of large-sized TiN inclusions persist, which pose significant detrimental effects on material performance. To address this critical issue, process optimization strategies such as enhanced cooling rates or reduced titanium content must be implemented to effectively suppress the formation of these deleterious large-scale TiN precipitates.
3. The number density of TiN inclusions at the center position of the slab is significantly lower than that at the quarter-thickness position. At both the quarter-thickness and center positions of the slab, the number density of TiN inclusions exhibits a gradual decrease from the edge to the interior regions in the samples. At the quarter-thickness of the slab, the highest number density is 40.3 mm^−2^ and the lowest number density is 34.2 mm^−2^. At the center of the slab, the highest number density is 41.3 mm^−2^ and the lowest number density is 13.4 mm^−2^.
4. The calculation results demonstrate that in high-titanium steel containing 0.4% Ti, TiN inclusions begin to precipitate in the liquid phase; reducing the titanium content in the steel can effectively delay the precipitation of TiN. During solidification, TiN continuously precipitates with progressive size increases as solidification proceeds. Notably, the size of TiN inclusions decreases with increasing cooling rates. When the cooling rate is 0.5 K/s, the maximum size of TiN inclusions reaches 6 μm, whereas at a higher cooling rate of 10 K/s, the maximum size decreases significantly to 1.3 μm.

## Figures and Tables

**Table 1:** The main elemental content of the high-titanium steel, mass%.

Element	C	N	Si	Mn	S	P	Mo	Ti	Al
Content	0.18	0.004	0.22	1.3	0.015	0.02	0.22	0.40	0.04

**Table 2:** Interaction coefficients of elements at 1600 °C [38,39,40].

I	e_i_^C^	e_i_^Si^	e_i_^P^	e_i_^S^	e_i_^Al^	e_i_^N^	e_i_^Mn^	e_i_^Ti^
Ti	−0.165	0.05	−0.11	−0.006	0.035	−1.8	0.0043	0.013
N	0.13	0.047	0.045	0.007	−0.028	0	−0.02	−0.53
C	0.14	0.008	0.051	0.046	0.043	0.11	−0.012	
S	0.11	0.01	0.029	−0.028	0.035	0.01	−0.026	−0.072

## Data Availability

The original contributions presented in this study are included in the article. Further inquiries can be directed to the corresponding author.
